# Effect of Intravenous IgM-Enriched Immunoglobulins on Presepsin and Other Sepsis Biomarkers

**DOI:** 10.3389/fphar.2021.717349

**Published:** 2021-09-08

**Authors:** Giuliana Scarpati, Daniela Baldassarre, Giovanni Tripepi, Massimo Boffardi, Ornella Piazza

**Affiliations:** ^1^ Dipartimento di Medicina e Chirurgia, Università Degli Studi di Salerno, Baronissi, Italy; ^2^ AOU San Giovanni di Dio e Ruggi D’Aragona, Salerno, Italy; ^3^ Section of Biostatistics, Institute of Clinical Physiology, CNR-IFC, Reggio Calabria, Italy

**Keywords:** presepsin (soluble CD14-subtype), sepsis - diagnostics, septic shock (MeSH), immunoglobulin M, elderly

## Abstract

Patients in septic shock with low IgG and IgM serum concentrations have higher mortality rates compared to those with normal immunoglobulin levels and, therefore, there is a rational explanation to administer intravenous IgM-enriched immunoglobulins to septic patients in ICU. Aim of this study is to evaluate the effectiveness of intravenous IgM-enriched immunoglobulins in decreasing several sepsis biomarker concentrations. 26 sepsis patients were enrolled in this observational cohort study and Nitric Oxide, Endocan, Pentraxin and presepsin serum levels were measured during their first 3 days of ICU stay. The use of intravenous IgM-enriched immunoglobulins did not influence the temporal evolution of SOFA, Nitric Oxide, Endocan, Pentraxin and Presepsin in the first 3 days of ICU stay in a statistically significant manner, even if Presepsin decreased of 25% from day 1 to day 2 in the Pentaglobin group. It seems possible that Pentaglobin infusion reduces the Presepsin level in a more effective way if it were administered to a younger population (*p* = 0.012). In conclusion, age modifies the response of Presepsin to Pentaglobin and is a critical variable when investigating the effect of intravenous IgM-enriched immunoglobulins on sepsis.

## Introduction

Elderly patients are predisposed to an increased rate of sepsis because of their state of immunosenescence and they suffer a more prolonged proinflammatory response compared to younger patients. Despite seniors have the capability to preserve antibodies against previously exposed antigens, they have a decreased ability to produce specific opsonophagocytic antibodies against neoantigens (new antigens) (Nasa et al., 2012).

Patients in septic shock with low IgG and IgM serum concentrations have higher mortality rates compared to those with normal immunoglobulin levels (Myrianthefs et al., 2010).

Many preparations, including intravenous IgM-enriched immunoglobulins, are available with a growing number of accepted uses, since these products are generally well-tolerated and with few side effects (Biagioni et al., 2021).

Polyclonal intravenous immunoglobulins have pleiotropic effects on inflammatory and immune mechanisms and have been proposed as adjuvant therapy to modulate inflammatory processes. During the SARS epidemic in Hong Kong, e.g., in selected cases, Tsui et al. (2003), and Ho et al. (2004) administered intravenous IgM-enriched immunoglobulins (Pentaglobin). One case of COVID-19 treated with polyclonal preparation of IgM as adjuvant therapy (Carannante et al., 2020) has been published.

Nevertheless, in 2016, Surviving Sepsis Campaign guidelines suggested against immunoglobulins use in sepsis because the available evidence was not clearly sufficient to support their widespread use in the treatment of sepsis (Rhodes et al., 2017). However, there is new experimental evidence (Vaschetto et al., 2017) supporting the rationale for IgM enriched immunoglobulin solution use in sepsis patients, and at the same time, no specific drug or strategy up to now has proven to be efficacious in reducing all causes sepsis mortality. Results from recent trials not included in the Cochrane metanalysis used by the SSC expert in the 2016, indicate that intravenous IgM-enriched immunoglobulins may be effective in septic patients (Cavazzuti et al., 2014; Giamarellos-Bourboulis et al., 2016). Cui et al. (2019) in a meta-analysis published in 2019, including 19 studies (1,530 patients), estimated that mortality rates were significantly lower in patients who received intravenous IgM-enriched immunoglobulins than in their respective control groups [relative risk (RR) 0.60; 95% confidence interval (CI) 0.52–0.69]. An ongoing trial (Biagioni et al., 2021) aims to discriminate if a personalized dose based on patients serum IgM versus standard dose is more efficacious in the treatment of septic shock (IgM-fat trial registered on ClinicalTrials.gov (NCT04182737) on December 2, 2019).

On the other hand, the search for reliable and precocious biomarkers of sepsis is never ending: procalcitonin and presepsin are widely used to diagnose sepsis and guide antimicrobial therapy, but the list could include also other molecules which may be relevant to understand the physiopathology of sepsis, as Nitric Oxide (NO), Endocan, Pentraxin (Supplementary material file). We aimed to prove the efficacy of intravenous IgM-enriched immunoglobulins by using indirect indicators of outcome; our hypothesis is that treated patients should benefit of a lower level of sepsis biomarkers (presepsin, endocan, pentraxin, plasmatic Nitric Oxide), compared to not treated subjects.

## Methods

The protocol for this study was reviewed and approved by the Ethics Committee Campania Sud as no profit research (protocol N. 165/2018).

Twenty-six consecutive patients with sepsis and septic shock were admitted to the University of Salerno ICU and enrolled in this observational cohort study. Based on the decision of the physician in charge, patients received an intravenous immunoglobulin preparation (Pentaglobin) in addition to standard therapy, or received standard therapy only (fluids, vasopressors, antibiotic therapy, parenteral and/or enteral nutrition) and were considered as the control group. The treatment with Pentaglobin for patients with septic shock was chosen always according to the physicians judgment, with exclusion of those presenting hypersensitivity to the IgM enriched preparation in use or its excipients, or had previous intravenous immunoglobulin therapy, selective absolute IgA deficiency with antibodies to IgA, pregnancy or breastfeeding or a positive pregnancy test. We did not include patients for whom clinical decision to withhold life-sustaining treatment was taken or when the diagnosis of shock was uncertain. Pentaglobin therapy started on the same day sepsis was diagnosed; 5 ml/kg Pentaglobin per day was infused for 72 h. Left over blood samples for NO, Pentraxin and Endocan were acquired daily for 3 days, the first sample was collected before the IgM enriched solution infusion and the third was collected after its end. After centrifugation at room temperature using 2500 G-force for 10–15 min, serum was transferred in 1–2 ml aliquots tubes, which were stored at −80°C until assayed by using a commercial solid-phase enzyme-linked immunosorbent assay (ELISA).

During the 1-year study period, presepsin was part of the routine clinical monitoring, measured by a commercial instrumentation on whole blood (PATHFAST Presepsin chemiluminescent enzyme immunoassay). Severity of critical illness and development of organ failure were assessed daily by sequential organ failure assessment (SOFA) score.

### Statistical Analysis

Data were summarized as mean and standard deviation or as median and interquartile range and the between-groups comparisons were performed by independent T-Test or Mann-Whitney Test, as appropriate. The between-groups differences (Pentaglobin treated patients versus controls) of the biomarkers evolution over time were investigated by linear mixed models (LMM) analysis. The effect modification by age on the difference in presepsin levels between Pentaglobin treated and untreated patients was investigated by the effect modification analysis, i.e. by introducing into the same multiple LMM (with presepsin as dependent variable), age (in years), treatment (1 = pentaglobin; 0 = no pentaglobin) and their interaction term (age x treatment). Given the fact that methodologists recommend to include into a multiple regression model 1 variable every approximately 5-9 patients (Vittinghoff and McCulloch, 2007), the optimal sample size requested for including the three variables mentioned above (that is, age, treatment, and age x treatment) was 26 patients. Such a sample size allows to adjust for 3 variables, i.e., 1 variable every approximately 9 enrolled patients. In the multiple LMM, age was considered either as confounder or as an effect modifier on the basis of clinical consideration. The difference in presepsin levels between Pentaglobin treated and untreated patients at pre-defined values of age was calculated by the linear combination method which allows to calculate the effect of Pentaglobin by assuming all the patients as having a specific age (40, 50, 60, 70, and 80 years). Data analysis was performed by SPSS for Windows version 22, IBM, Chicago, Illinois, United States.

## Results

15 septic shock patients were treated with Pentaglobin (males 40%) and 11 patients were treated with standard therapy (males: 45%). The characteristics of the study population are summarized in Table 1. The decision to treat with Pentaglobin was left to the physician in charge, independently from the purpose of this research. Included patients had a median age of 66 years (interquartile range 60–81); median SOFA score at ICU admission was 7 (interquartile range 5–11); median SAPS was 25 (interquartile range 17–35). Physicians decided to administer Pentaglobin to a more severe subset of patients: mean (and SD) SOFA score at admission was 18.6 ± 4.5 in Pentaglobin treated patients versus 9.5 ± 4.0 of the control group, but this difference was not statistically significant (*p* = 0.09). There was no substantial age difference between Pentaglobin and control group [mean age was 65 ± 16 (SD) years in Pentaglobin group and 71 ± 16 (SD) years in the control group; *p* = 0.35].

**TABLE 1:** Patients' characteristics and Predisposition, Infection/injury type, Response and Organ dysfunction (PIRO) score. The PIRO score includes eight variables, each given the same weight (Age, Comorbid conditions, Leukopenia, Hypothermia, Cardiovascular dysfunction, Respiratory dysfunction, Renal dysfunction, Central nervous system dysfunction).

	*n*. patients	Age (media)	Male	Female	Dead/alive	Predisposition	Infection (lung/abdomen)	SAPS II
Controls	11	71 ± 16	5	6	9/2	4	2/9	2728
Pentaglobin group	15	65 ± 16	6	9	9/6	10	3/12	2946

### Effect of Pentaglobin on the Temporal Evolution of SOFA, NO, Endocan and Presepsin

**FIGURE 1:**
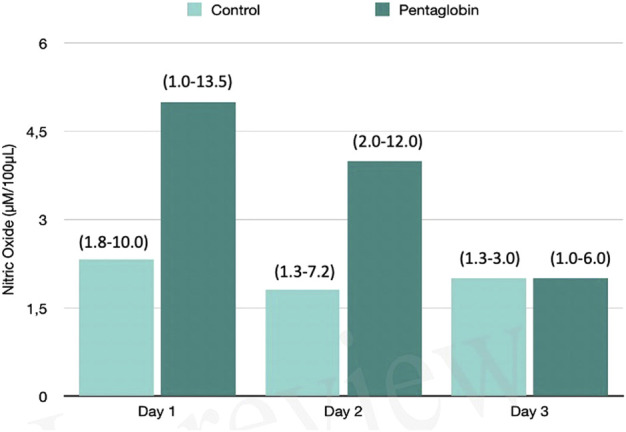
Comparison of Nitric Oxide changes over time between Pentaglobin and control groups by linear mixed models (*p* = 0.83). The height of bars represents the median values and the numbers in brackets the corresponding interquartile range of Nitric Oxide (NO) levels determined by ozone-chemiluminescence technology in blood samples in patients receiving intravenous immunoglobulin preparation (Pentaglobin) and in those of the control group. NO was collected for the first 3 days from sepsis diagnosis after ICU admission. The use of Pentaglobin did not significantly influence the temporal evolution of NO (*p* = 0.83) versus the standard therapy, although a decreasing trend of NO level at the end of the third day by more than 50% was observed in the treated group compared to the control group. The between-groups comparison over time was performed by linear mixed models. See text for more details.

**FIGURE 2:**
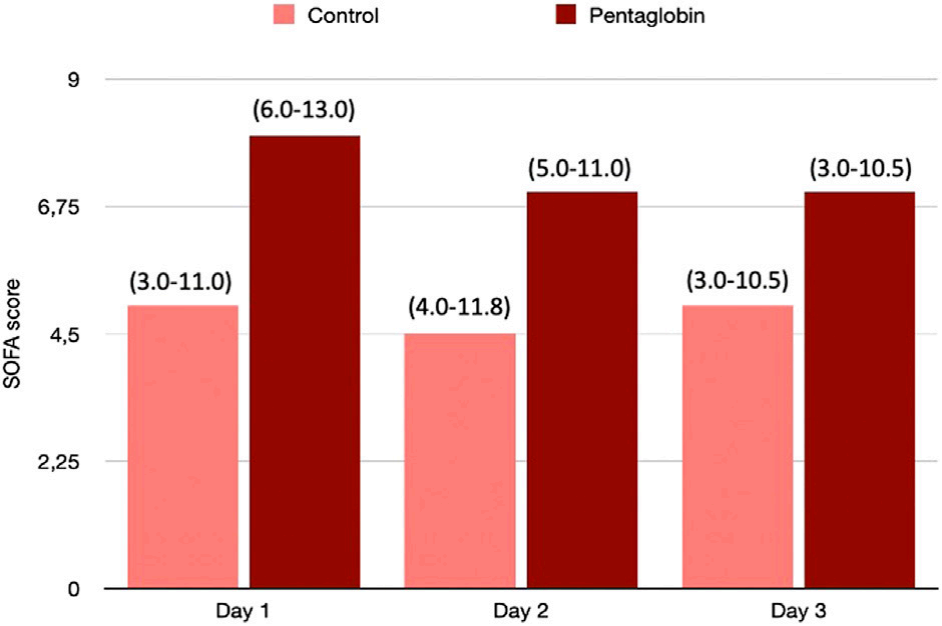
Comparison of SOFA changes over time between Pentaglobin and control groups by linear mixed models (*p* = 0.49). The height of bars represents the median values and the numbers in brackets the corresponding interquartile range of SOFA scores calculated daily to assess the severity of critical illness and the development of organ failure in Pentaglobin treated patients during the first 3 days of treatment after sepsis diagnosis compared with the control group. There was no significant difference in the SOFA score at admission between the Pentaglobin group and controls (*p* = 0.09). Furthermore, the use of Pentaglobin did not influence the temporal evolution of SOFA (*p* = 0.49) versus the standard therapy. See text for more details.

**FIGURE 3:**
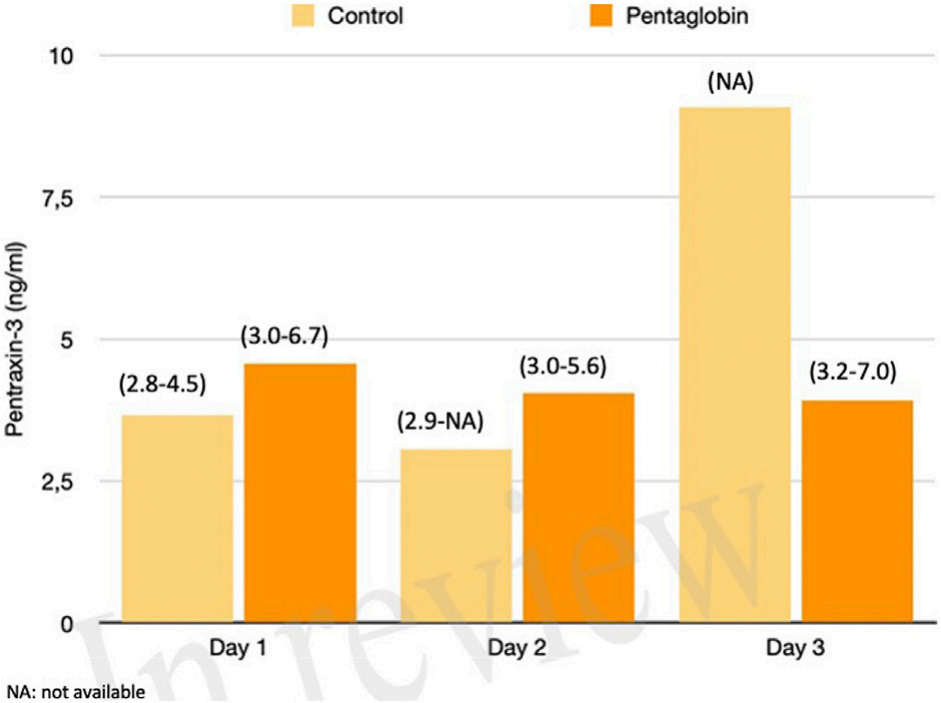
Comparison of Pentraxin-3 changes over time between Pentaglobin and control groups by linear mixed models (*p* = 0.96). The height of bars represents the median values and the numbers in brackets the corresponding interquartile range of Pentraxin-3 levels between Pentaglobin treated patients and controls during the first 3 days of ICU stay from sepsis diagnosis. PXT-3 levels increased daily up to the third day in septic patients treated with standard therapy (i.e., the control group) in agreement with the role of this acute-phase protein in the early phase of sepsis. The between-groups comparison over time was performed by linear mixed models and resulted to be not significant (*p* = 0.96). See text for more details.

**FIGURE 4:**
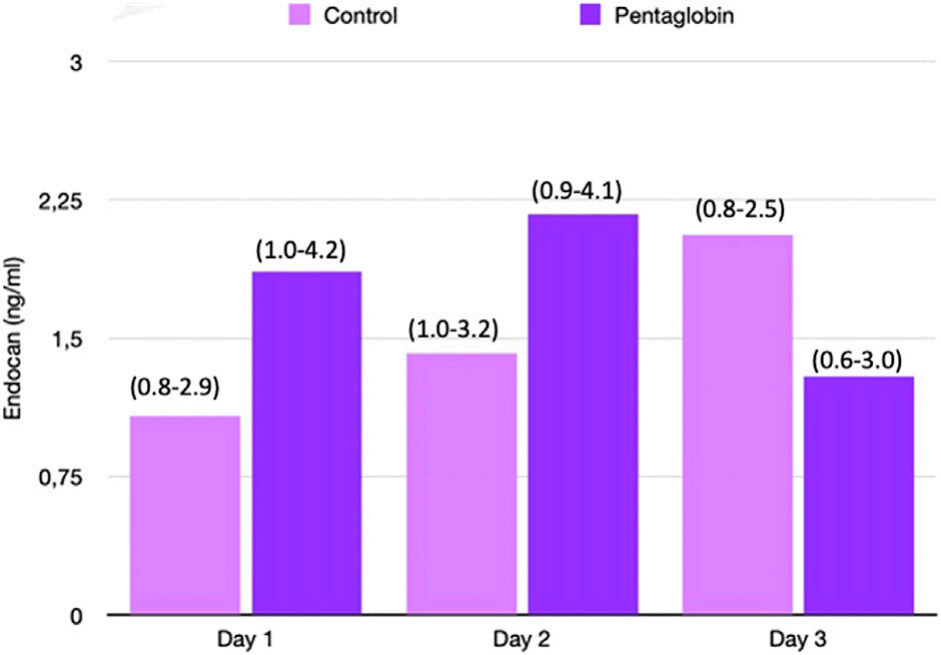
Comparison of Endocan changes over time between Pentaglobin and control groups by linear mixed models (*p* = 0.38). The height of bars represents the median values and the numbers in brackets the corresponding interquartile range of Endocan in Pentaglobin treated patients and in controls in the first 3 days from ICU admission and septic shock. Pentaglobin therapy seems to restrain a regular upward trend of the Endocan levels, which instead characterizes the first 3 days of septic patients who were only on conventional therapy. The between-groups comparison over time was performed by linear mixed models and resulted to be not significant (*p* = 0.38). See text for more details.

**FIGURE 5:**
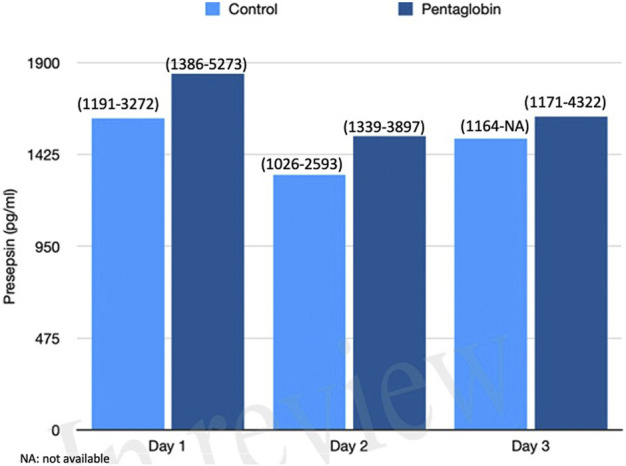
Comparison of Presepsin changes over time between Pentaglobin and control groups by linear mixed models (*p* = 0.20). The height of bars represents the median values and the numbers in brackets the corresponding interquartile range in Pentaglobin treated patients and in controls. Presepsin was measured for the first 3 days from diagnosis of septic shock and the figure shows decrease in its concentration of 25% from day 1 to day 2 in the Pentaglobin group, whilst the decrease was slightly lower in the control group. The between-groups comparison over time was performed by linear mixed models and resulted to be not significant (*p* = 0.20). See text for more details.

The use of Pentaglobin did not influence the temporal evolution of SOFA (*p* = 0.49), NO (*p* = 0.83), Endocan (*p* = 0.38), PTX (*p* = 0.96) and presepsin (*p* = 0.20) in the first 3 days of ICU stay (Figures 1–4). However, Presepsin, decreased by 25% from day 1 to day 2 in the Pentaglobin group, whilst the decrease was slightly lower in the control group (22%). The between-groups difference was not statistically significant (Figure 5).

### Effect Modification by Age on the Relationship Between Pentaglobin and Presepsin

**FIGURE 6:**
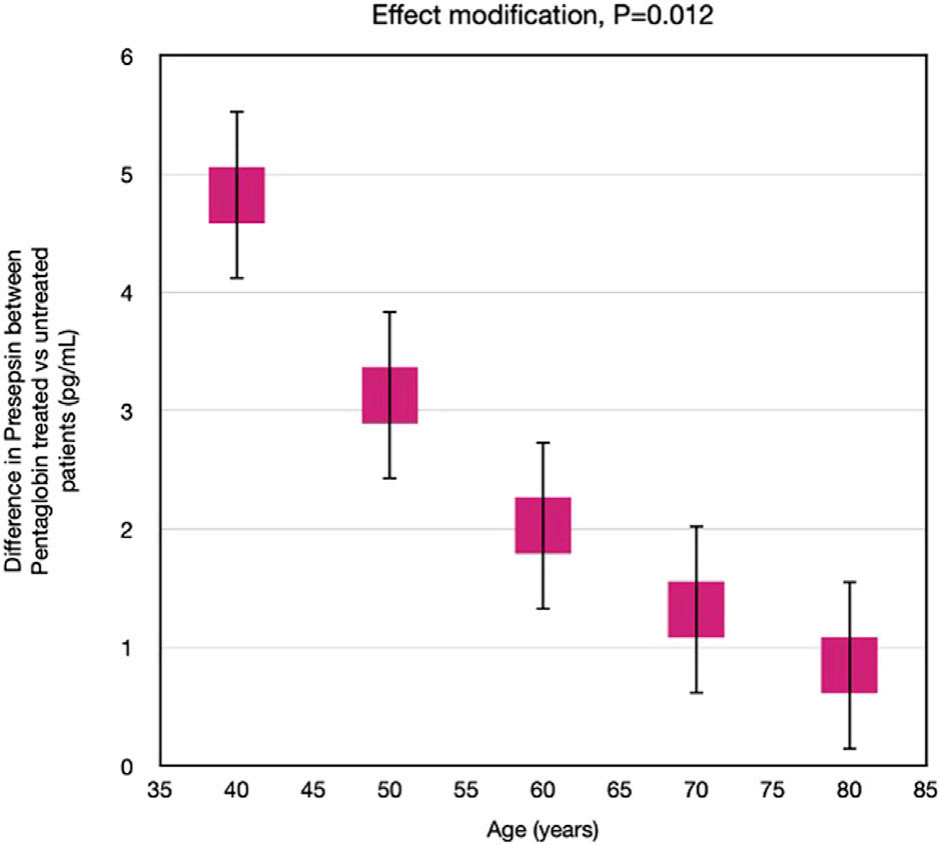
Effect modification by age on the effect of Pentaglobin on presepsin. The effect of Pentaglobin for increasing Presepsin, compared to the control group, was strictly dependent on age. Through an advanced statistical technique (linear mixed models), the effect of Pentaglobin assuming that all the patients in the study were of a certain age was estimated (such an approach allows to overcome the problem of the reduced sample size in the various age groups). If all patients were 40 years old, the difference in presepsin (Pentaglobin treated patients versus controls) would be 4.82, if they were 50 years old the difference in presepsin would be 3.13, if they were 60 years old the difference in presepsin would be 2.03, if they were 70 years old the difference in presepsin would be 1.32, if they were 80 years old the difference in presepsin would be 0.86. Therefore, the reduction of presepsin determined by Pentaglobin is greater the lower is the age. The difference in the effect of the drug between the various ages is statistically significant (*p* = 0.012). Data are mean differences and 95% CI.

Nevertheless, by applying linear mixed models, we calculated the Pentaglobin effect by assuming that all the patients had the same age, to overcome the limited number of patients in each decade. If all patients had had 40 years old, the difference in presepsin concentration between pentaglobin treated and control patients would have been higher rather than in patients of 50, 60, 70 or 80 years of age. In other words, age significantly modified (*p* = 0.012) the presepsin response to pentaglobin (Figure 6), the difference in presepsin levels between Pentaglobin treated and untreated patients being closely dependent on age. In fact, the higher the age, the lower the difference in presepsin levels between Pentaglobin treated and untreated patients.

## Discussion

The incidence of sepsis and septic shock is increasing in the elderly. They are predisposed to sepsis, due to the effects of aging itself and to the sum of co-morbidities, prolonged hospitalizations, functional limitations. Management is largely based on standard international guidelines while there is evidence that several drugs, i. e., antibiotics, need special care, since the pharmacokinetic parameters changes with aging and the side effects can differ. In the elderly, serum IgM concentrations are significantly reduced, whereas serum IgA concentrations are maintained (Lock and Unsworth, 2003). Nevertheless, no significative effect of Pentaglobin on sepsis biomarkers in elderly patients was detected, while statistical analysis addressed the greater potential benefit in a younger group. In literature, adverse effects from intravenous immunoglobulins have been reported and can be classified into three types according to their onset: immediate, delayed, and late onset. Immediate adverse effects occur during infusion, for example anaphylactoid reactions; delayed adverse effects occur hours or days after infusion, for example pulmonary, renal, haematologic and neurologic events; and late adverse effects include transmission of infectious agents such as hepatitis C and prion diseases (Nydegger and Sturzenegger, 1999). The present research did not report any adverse event related to the use of Pentaglobin. The surviving sepsis campaign (SSC) guidelines (Rhodes et al., 2017) suggest early eradication of septic foci, administration of anti-infective agents, maintenance of hemodynamic stability through fluid administration and vasopressors. The SSC guidelines remain the cornerstone of treatment for sepsis and the fact that in 2016, SSC guidelines suggested against Ig use in sepsis, because of weak evidence of efficacy from previous studies, surely reduced the number of treated patients in our ICU, discouraging physicians to prescribe a drug which is safe but not surely helpful. However, results from successive trials (Giamarellos-Bourboulis et al., 2016) and systematic meta-analyses (Cui et al., 2019) indicate that intravenous IgM-enriched immunoglobulins reduced mortality (Cui et al., 2019) and the addition of IgM enriched solution to the SSC bundles appears as a promising treatment option, in particular in those patients with an acute disease onset, who are heavily inflamed, showing signs of overt septic shock and those with an immunocompromised phenotype, (patients in an immunosuppressive stage) (Nydegger and Sturzenegger, 1999; Nierhaus et al., 2020).

The main limitation of this study is the low sample size, even if we systematically collected all the sepsis shock patients admitted to our ICU during the study period. This pilot study needs a follow up, with a focused clinical trial, aimed to understand the role of age in IgM infusion efficacy.

## Conclusion

In this limited case series, we found no evidence that Pentaglobin has a direct and significant effect on the investigated sepsis biomarker levels. Nevertheless, age modifies the response of Presepsin to Pentaglobin and can be considered as a critical variable whilst investigating the effect of intravenous IgM-enriched immunoglobulins on sepsis.

## Data Availability

The raw data supporting the conclusion of this article will be made available by the authors, without undue reservation.
