# Supplementary material for: Effect of Intravenous IgM-Enriched Immunoglobulins on Presepsin and Other Sepsis Biomarkers
Source: Front Pharmacol. 2021 Sep 8;12:717349. doi: 10.3389/fphar.2021.717349 (PMC8455986; doi:10.3389/fphar.2021.717349)
Supplement: Supplementary file 1 [file DataSheet1.docx]

Supplementary Material

1. Nitric Oxide

Excessive production of nitric oxide (NO) in sepsis, following the induction of iNOS, is due to the increased circulating cytokine concentration, bacteria and microbial products, acting in strong synergy. The conversion of l-arginine to NO by the NO synthases and subsequent degradation of NO into nitrite and nitrate is part of the adaptive response of the host defence mechanism, since NO has bactericidal effects.

For this study, NO was determined by a gas phase chemiluminescence reaction of NO with ozone using a Sievers Nitric Oxide Analyzer (NOA, GE Analytical Instruments) as already described elsewhere (Nagababu E, Rifkind JM. Measurement of plasma nitrite by chemiluminescence without interference of S-, N-nitroso and nitrated species. Free Radic Biol Med. 2007;42(8):1146–1154. doi:10.1016/j.freeradbiomed.2006.12.029).

2. **Endocan**

Endothelial cell specific molecule-1 (endocan) is a proteoglycan mainly expressed by lung endothelial cells, whose secretion in the bloodstream is upregulated by proinflammatory cytokines (IL-1β, TNF-α) and bacterial LPS.

Endocan concentration can be used as predictor of sepsis severity and outcome (<https://doi.org/10.1016/j.mvr.2014.04.004>).

In this research, Endocan concentrations were quantified by a commercial solid-phase enzyme-linked immunosorbent assay (ELISA).

3. Pentraxin 3

PTX3 is an acute-phase protein. PTX3 plasma concentration increases rapidly in sepsis since it plays an important role in the early phase of inflammation, as it activates the classical pathway of

complement and contributes to the opsonization and clearance of apoptotic or necrotic cells (<https://doi.org/10.1371/journal.pone.0053661>.)

The PTX3 concentration was determined using a commercial solid-phase enzyme-linked immunosorbent assay (ELISA)

4. Presepsin

Among the newest acquisition in the critical care armament, Presepsin (Handke J, Piazza O, Larmann J, Tesoro S, De Robertis E. Presepsin as a biomarker in perioperative medicine. Minerva Anestesiol 2020;86:768-76. DOI: 10.23736/S0375-9393.20.14169-5) is released by monocytes after the recognition of bacterial lipopolysaccharide or other surface bacterial ligands including gram-positive peptidoglycans. Several studies documented the role of Presepsin in the diagnosis of sepsis, where higher levels correlated with severity.

Presepsin is derived from cleavage of CD14 (cluster-of-differentiation marker protein 14), a glycoprotein present on the membrane of monocytes, macrophages and polymorphic neutrophils
